# Level of exclusive breastfeeding and its enabling factors among lactating women who delivered in health facilities of Asosa town, Ethiopia: a cross sectional study

**DOI:** 10.1186/s12905-021-01580-2

**Published:** 2021-12-28

**Authors:** Megersa Kumera, Jemal Haidar

**Affiliations:** 1 Ethiopian Pediatric Society, Addis Ababa, Ethiopia; 2 College of Health Sciences, School of Public Health, Addis Ababa University, Addis Ababa, Ethiopia

**Keywords:** Lactating mothers, Exclusive breastfeeding practice score, Enabling factors, Ethiopia

## Abstract

**Background:**

Despite the enormous benefit of exclusive breastfeeding (EBF) to mothers and infants, the practice of exclusive breastfeeding is globally low. In sub-Saharan Africa and Ethiopia, the prevalence of EBF stands at 35% and 59%, respectively. The low EBF practice in Ethiopia as well as in the studied region calls for further study and thus we studied the EBF practice in the study area since little is known about its current magnitude and factors influencing its practice for some programmatic improvements.

**Methods:**

A facility based cross-sectional study linked to a community was conducted from June-July 2019 among 412 mothers who had antenatal follow-up and delivered in health facilities of Asosa town over a period of one year prior to the study. Data on socio demographic characteristics and other important variables were collected through face to face interview while gestational age in weeks was recorded from their medical chart by trained health extension workers in accordance with relevant ethical guidelines and regulations. The collected data were then cleaned and entered into Epi-data software version 3.02. Analysis was done by SPSS version 20. Binary and multivariate logistic regression were performed to identify the contributing factors. P-value of less than 0.05 and 95% confidence interval was considered to determine statistical significance.

**Results:**

Of the 412 respondents, the majority (88.1%) were multi-gravida and above. Slightly higher than a quarter (26.0%) and over half (55.4%) had neither received antenatal nor postnatal care. The proportion of mothers who exclusively breastfed their children was 76.0% and the overall aggregated good practice of EBF score was 64.1%. Mothers who completed primary school [AOR = 4.5; 95% CI = 1.1,18.2], had four or more ANC [AOR = 1.8;95CI = 0.79–0.98], and postnatal follow-up [AOR = 0.21;95% CI = 0.07–0.67], and had male infants [AOR = 2.3; 95% CI = 1.0–4.95] were among the factors influencing the exclusive breastfeeding practice score.

**Conclusion:**

While three in four mothers exclusively breastfed their newborns and about two-thirds had good EBF score, the observed women’s retention on the continuum of the maternal care pathway is low with one in four had no antenatal and over half had no postnatal care which are important derivers for EBF practice. To improve the EBF score and narrow the observed maternal and child health disparities, it is essential to promote maternal education and increase the recomended coverage of antenatal and postnatal care for mothers.

## Background

Exclusive breastfeeding (EBF) refers to provision of breast milk only to infants from their biological mothers or wet nurses’ expressed milk with the exception of medicines [1]. Feeding breast milk (BM) alone for the first six months of life is adequate because it contains all the necessary nutrient requirements in terms of quantity as well as quality in addition to its immunological properties which protecs the infants from frequent illnesses and improves his/her chance of survival [2].

It is widely documented that the first year of life is very central for all growing child in improving the quality of life and thus nature has provided them with a pefect natural food that contains all the essential nutrients needed for growth and development. To maximize the benefits obtained from BM, infants need to be fed on demand at least 8 times in 24 h [3]. Infants that are exclusively breastfed are often healthier than their counterparts during their first year of life and consequently retards their growth [4]. Other benfits documented are economical such as reducing healthcare costs [5, 6] improving maternal-child bonding, lowering the risk of developing non communicable diasese and beyond [7].

Breast milk is the healthiest form of milk for human babies with few exceptions like when the mother is taking certain drugs or is infected with tuberculosis or HIV [8]. Because of its enormous benefits, researchers advise the use of human milk to feed their babies, especially the initial milk, called colostrum, rich in immunoglobulin’s that coat the gastrointestinal tract and protect the newborns until their own immune system starts properly functioning and creates a mild laxative effect, expelling meconium and preventing the buildup of bilirubin [9].

The World Health Organization (WHO) and UNICEF strongly recommend mothers breastfeed their newborns within one hour after birth and continue doing so exclusively for the first six months of life [10, 11]. Despite the aforementioned recommendations, the proportion of mothers who practiced EBF was 52.0% in 2011, and 59.0% in 2016 based on the Ethiopian Demographic Health Survey reports [12, 13]. Although the 2016 survey report showed a slightly increment in the proportion of EBF, the practice declined with age from 74% in 0–1 months to 36% in 4–5 months with some regional variations. A more recent pocket study in rural Ethiopia reported EBF practice of 43.6% which is even lower than the 2016 national fidings [14]. It is important to note that the observed gap in EBF practice is not only due to lack of awarenesses-it is compounded by low physical access to health care facilities, economic, societal and cultural settings [15–18] are among other barriers. Many questions remain about the who, where, and how we could improve EBF, particularly for poor and marginalized populations, and how to strengthen the service in varying settings whilst linking to the health system. Most studies have so far been done elsewhere and available information in this regard is scanty for the studied region. Therefore this study attempted to generate evidences on the level of EBF practice and its enablers which are vital for the well being of infants.

## Methods

### Study area and population

A facility based cross sectional study linked to a community was conducted from June to July 2019 in Assosa town, the capital city of Benishangul-Gumuz (BG) among lactating mothers. BG is amongst the nine regional states of the Federal democratic republic of Ethiopia. It is an emerging region that has 3 zones and 20 districts for administrative purposes. It is located in western Ethiopia to the border of Sudan. It was previously known as Region 6. The capital town has one public hospital and one health center serving for over 600, 000 inhabitants. The study participants were all postnatal volunteer mothers that had antenatal follow-up and delivered in the aforementioned facilities during the study period.

### Sample size determination, data collection, processing and analysis

The sample size required was determined using single population proportion formula assuming a proportion of 34.7% of ogood practice of EBF score [19] with level of significance of 5% (α = 0.05), 95% confidence level (Z α/2 = 1.96) and absolute precision or margin of error of 5% (d = 0.05) and 10% for non-response rate which inflated the sample to 412. To allocate the required sample size for each facility, we initially included all mothers that delivered in both facilities during the preceding one year based on the medical chart (N = 1056). Then divded the total number (N) to the size of mothers who delivered in the health center (n = 206) and the hospital (n = 850) and subsequently distributed the required sample size proportionately to the health center and the hospital. Following this procedure, 80 (1056/206) were assigned for the health center and the rest 332 (1056/850) were allocated for the hospital. Once the allocation process was completed, the next step was to select and recruit all the eligible in the community with the help of the Health Extention Workers (HEWs) until the required sample size was reached.

To standardize the data collection process, 2 days training was given for five data collectors (HEWs) and one BSc Nurse supervisor on data collection techniques and work ethics. Data were then collected on sociodemographic, obstetric factor, neonatal factor and culture related variables from all sampled participants through face to face interview using a pre-tested structured tool adapted from similar study localy while gestational age in weeks was recorded from their antenatal follow-up chart. Six practice questions were included in the tool to assess practice of EBF and distributed as; (1) breastfeed your last child (2 items); (2) initiation of breastfeeding (3 items); (3) frequency of BF (3 items); (4) substance given before initiating BF (2 items); (5) types of Prelacteal feeding (4 items) and (6) child BF from 0–6 months only breast milk (2 items). The items in each construct were then added together, with equal weights, to generate the mean score and then categorized the collected information into good and poor EBF practices when the overall mean scores of is above and below the mean, respectively.

Data were entered and analyzed using SPSS software version 25.0. Bivariate logistic regression analysis was used to assess the degree of association between good EBF practice and its contributory factors and test significance of the association. In addition, variables which had significant association with the outcome variable were entered into multivariate analysis model, to identify the important determinants and used to control for possible confounding effects. Those tested variables with a significance level of 20% in the bivariate analysis were further entered in the multivariate logistic regression model to identify the predictors that retained their association with the outcome. A p-value less than 5% was declared as statistically significant.

## Results

### Sociodemographic characteristics

All the 412 enrolled mothers were interviewed and responded adequately with 100% response rate. The proportion of mothers belonging to the age of 26-35 years was 70.1% and 165 (40%) were Orthodox Christians. Less than a quarter (20.1%) were unable to read and write and about one-third (30.3%) were housewives. About half (47.6%) were from rural settings and most (76.5%) of them were earning below 5000 ETB 52.4 (Table 1).

**Table 1:** Respondents sociodemographic characteristics

Respondents characteristics	Number	Percent
*Age of the mother*		
< 25 years	82	19.9
26–35 years	289	70.1
36–45 and above	41	10
*Religion of the mother*		
Orthodox	165	40.0
Muslim	124	30.1
Catholic	41	10.0
Protestant	82	19.9
*Marital status*		
Married	306	74.3
Single	33	8.0
Divorced	65	15.8
Widowed	8	1.9
*Educational status of the mother*		
Unable to read and write	83	20.1
Read and write	163	39.6
Primary school	58	14.1
Secondary school	52	12.6
10 + 2 and above	56	13.6
*Maternal occupation*		
Government employee	124	30.1
Private employee	163	39.6
House wife	125	30.3
*Residency*		
Urban	216	52.4
Rural	196	47.6
*Income*		
< 1500	154	37.4
1500–2500	71	17.2
2501–5000	90	21.8
> 5000	97	23.5

ETB-Ethiopian Birr (1 USD is equivallen to 39 ETB at the time of the study)

### Maternal obstetrics and newborn characters

Of the 412 respondents, the majority (88.1%) were multi-gravida and above. Slightly higher than a quarter (26.0%) had no antenatal care. The proportion of mothers who had three and four ANC was 40.3% and 21.6%, respectively. Over half (55.4%) had no postnatal care showing women’s retention on the continuum of the maternal care pathway is low. The proportion of mothers who had normal range of gestational age in weeks, low birthweight and exclusively breastfed their children was 79.9%, 12.2% and 76.0%, respectively (Table 2).

**Table 2:** Maternal obstetrics and newborn characters

Variables	Number	Percent
*Gravidity*		
Primigravida	49	11.9
Multigravida	245	59.5
Grand multipara	118	28.6
*Antenatal care (ANC)*		
None	111	26.9
only one times	43	10.4
Twice	3	0.7
Thrice	166	40.3
Four and more	89	21.6
*Postnatal care (PNC)*		
Yes	184	44.7
No	228	55.3
*Gestational age (GA)*		
32–37 weeks	83	20.1
37–42 weeks	329	79.9
*Child birthweight*		
< 2500 g	71	12.2
2500–4000 g	176	42.7
> 4000 g	85	20.6
Unknown	80	19.44
*Breastfed the child exclusively*		
Yes	313	76.0
No	99	24.0

GA was recorded in weeks from their antenatac follow-up card

### Maternal practice towards exclusive breastfeeding

Table 3 displays the maternal experiences towards EBF. Most (70.1%) of the mothers breastfed their infants and 248 (60.2%) initiated BF immediately, and over one-third (39.8%) fed their infants regularly ever 3–4 h. Eighty two (19.9%) had provided pre-lacteal feeding of whome, 12.3% gave plain water, cow’s milk in 10.9%, butter in 24.4% and formula milk in 52.4%. The overall aggregated good EBF practice score was 64.1%,

**Table 3:** Maternal practice towards exclusive breastfeeding

Characteristics	Number	Percent
*Breastfeed the infant*		
Yes	289	70.1
No	123	29.9
*Initiation of breastfeeding after delivery*		
Immediately	248	60.2
Between 2 and 24 h	123	29.9
After 24 h	41	10.0
*Frequency of breastfeeding*		
On demand	124	30.1
Regularly every 3–4 h	164	39.8
Randomly	124	30.1
*Pre-lacteal feeding*		
Yes	82	19.9
No	330	80.1
*Types of pre-lacteal feeding provided (n* = *82)*		
Plain water	10	12.3
Cow milk	9	10.9
Butter	20	24.4
Formula milk	43	52.4
*Since birth to 6 months child was given*		
Breast milk only	313	76.0
Formula milk	99	24.0
*Overall practice of EBF score*		
Good	264	64.1
Poor	148	35.9

### Factors affecting good EBF practice

Prior to the regression analysis, maternal responses on the six dichotomized practice questions were fit into the binary regression model. The output revealed that mothers that had education, from urban setting, had antenatal and postnatal follow-up, and gave birth to male child were significantly associated with practicing good EBF. Nonetheless, in the multivariate regression analysis, only mothers who had completed primary schooling [AOR = 4.5; 95% CI = 1.1,18.2], had four and more ANC [AOR = 1.8;95CI = 0.79–0.98], postnatal follow-up [AOR = 0.218;95% CI = 0.07–0.67], and delivered male infant [AOR = 2.3; 95% CI = 1.0–4.95] retained their significant association with good exclusive breastfeeding practice score. Although more mothers that had received information from health facilities adhered to good EBF, there was no significant association with the EBF practice (Table 4).

**Table 4:** Maternal and child factors contributing to practice exclusive breastfeeding

Characteristic	EBF score		COR95% CI	AOR95% CI
	Good	Poor		
*Education status of mother*				
Unable to read and write	9	42	1	1
Read and write	39	87	2 (0.93–4.7)	0.26 (0.06–1.02)
Primary school	96	19	23.6 (9.8–56.4)*	4.5 (1.1–18.2)*
Secondary school	49	57	4 (1.8–9.06)*	0.54 (0.13–2.35)
10 + 2 and above	13	1	60 (7.01–524.8)*	8.5 (0.77–94.3)
*Residency*				
Urban	133	83	2.7 (1.8–4.02)*	2.3 (1.17–04.56)
Rural	73	123	1	1
*Frequency of ANC follow up*				
None	18	93	1	1
One	3	40	0.12 (0.06–0.23)*	0.14 (0.06–0.36)*
Twice	0	3	0.046 (0.013–0.16)*	1.7 (0.86–3.5)
Three	130	36	1.9 (1.4–3.66)*	1.8 (0.13–0.18)*
Four and above	55	34	2.0 (1.8–3.4)*	1.8 (0.79–0.98)*
*Infant gender*				
Female	80	111	1	1
Male	126	95	0.54 (0.36–0.8)*	2.3 (1.0–4.95)*
*Received PNC*				
Yes	105	166	2.4 (1.55–3.55)*	0.22 (0.07–0.67)*
No	45	88	1	1
*Source of information*				
Friends	44	41	1	1
Mass media	48	80	0.55 (0.32–0.97)	1.7 (0.6–5.4)
Facilities	114	85	1.25 (0.75–2.18)	0.7 (0.29–1.75)

*Denotes significance (p < 0.05); COR = Crude odds ratio; AOR = Adjusted odds ratio

## Discussion

The world health organization adopted a comprehensive strategy called the Continuum of Care to improve reproductive, maternal, neonatal, and child health [20, 21]. In view of this, we studied the practice of EBF and its enabling factors among mothers who had their antenatal follow-up and delivered over a one year period in the two available health facilities linked to the community. Based on our findings, about three-fourth adhered to EBF which indicates that present finding is better than the the 2016 national figure reported by Ethiopian Demographic Health Survey report [13] which was 59%. It is likely to see such discrepancies since the nature of the sampled mothers enrolled in our study were from health facilities suggesting that all had probably accessed and utilized the reproductive health services that included counselling on breastfeeding and to some extent might had information about the importance of EBF.

Though breastfeeding is almost universal in Ethiopia and exclusive breastfeeding up to 6 months after birth is an important contraceptive method, the status of exclusive breastfeeding in the studied region as well as in the country is less than the global recommendations [22] still more focused advocacy work needs to be done in this regard.

The good practice when compared with previous research reports, our study is concordant with Mizan-Aman town findings which reported 60.2% of the mothers to initiate breastfeeding immediately within an hour (60.2% vs 60.1%). The similarity of the findings could be attributed to the fact that all the sampled mothersin both the studies are from health facilities where they had access to appropriate councelling on newborn feedings and other reproductive health services [19, 23]. Although the present finding is relatively better than other emrging regions in the country, further work is needed to achiev a better coverage of EBF and maximize its health benefites for the child as well as the mothers.

In this study, pre-lacteal feeding (PF) observed is higher compared to the findings reported by other authors [18, 22–24]. The differences may be attributed to time constrains emanating from work related activities—most mothers with better education often get better job with better income and often such mothers rush to their work and consequently forced to practice PF. There could be also other reasons which requires further expoloratory study. The most common PF given in this study was plain water, cow’s milk, butter and formula milk. Compared with the Mizan Aman findings on provision of water (12.3% vs 48.2%), and cow’s milk (24.4% vs 50.2%), our finding is better and the difference might be due to socioeconomic factors and to some extent to maternal awareness towards EBF though the assertion needs further study.

The major enabling factors to good EBF practice score were education, ANC of four and more and infant’s gender while postnatal care had an inverse relation. Mothers who had primary education were 4.5 time more likely to adher to EBF than their counterparts. Although the education system differs and various experiences exist across the world, studies have reported maternal and paternal educational attainment as a common predictor for maternal health service utilization in developing countries including Ethiopia [25, 26].

Mothers who had four or more antenatal follow-up were 1.8 times more likely practicing EBF than those who had none. Surprisingly, having postnatal follow-up in this study showed an inverse relation with EBF. This observation however, needs to be interpreted carefully since PNC is an opportunity for nutrition/EBF promotion. It appears that the low coverage of PNC could have negatively influenced the EBF practice along the continuum of care and found to be among the weakest of all reproductive and child health programmes in the region as well as elsewhere among rural women due to the high domestic workload and little time left after attending to essential household chores until term and beyond [27, 28]. To ensure a seamless continuum of care from home to hospital and most importantly good EBF practice, the PNC program needs to be strengthened accros the region as well as in the country.

Interestingly, mothers who delivered male infant were observed to practice 2.3 times more likely than those who delivered females probably due to cultural preferences for males. Other noteable finding of the present study is that mothers that had received information from health facilities adhered to EBF though the association with the EBF practice was not significantly probably due to an unfocussed councelling. This is in contrary to some previous studies where they reported some positive associations and thus the observation needs cautious interpretations [15, 26] and warrants for further exploratory study to uncover why the sources of information did not bring the desired/expected change toward EBF practice.

## Strength and limitations

The strength of the study is that it included the available health facilities in the town and has generated some new evidence for programmatic implications to improve the EBF practice in the region. The information generated would also serve as a bench mark for future studies. Despite the aforementioned strengths, the findings had some limitations and thus need to be interpreted cautiously since facility-based studies as well as the design employed may make estimates unstable and associations between dependent and independent variables undetectable.

## Conclusion

The study has demonstrated that three in four mothers were practicing EBF with an overall good EBF practice score of 64.1%. The enabling factors identified are having education and attending the recommended antenatal care of four and above as well as delivering male infants. While the EBF practice is better than the national figure, the observed women’s retention on the continuum of the maternal care pathway is low with one in four to have no antenatal and over half had no postnatal care which are critical to the health and survival of a mother and her newborn. To narrow the disparities observed among mothers and their children, it is essential to intervene through focused actions that address the above determinants by all stakeholders since the issue is a cornerstone of public health and it has been reiterated at many international conferences and initiatives to lower morbidities and mortlities of mothers and children [28, 29].

## Data Availability

All data generated or analysed are included in this published article.

## Abbreviations

ANC: Antenatal care
AOR: Adjusted odds ratio
BG: Benishengul-Gumuz
BM: Breastmilk
EBF: Exclussive breastfeeing
ETB: Ethiopian birr
PF: Prelacteal feedings
PNC: Postnatal care
